# A Mechanistic Model for Hydrogen Production in an AnMBR Treating High Strength Wastewater

**DOI:** 10.3390/membranes13110852

**Published:** 2023-10-25

**Authors:** Gino Vera, Felipe A. Feijoo, Ana L. Prieto

**Affiliations:** 1 Department of Civil Engineering, Universidad de Chile, Santiago 8380453, Chile; 2 School of Industrial Engineering, Pontificia Universidad Católica de Valparaíso, Valparaíso 2340000, Chile; 3 Advanced Center for Water Technologies (CAPTA), Universidad de Chile, Santiago 8370449, Chile

**Keywords:** AnMBR model, multi-substrate model, membrane fouling, fermentative hydrogen, wastewater-to-H_2_

## Abstract

In the global race to produce green hydrogen, wastewater-to-H_2_ is a sustainable alternative that remains unexploited. Efficient technologies for wastewater-to-H_2_ are still in their developmental stages, and urgent process intensification is required. In our study, a mechanistic model was developed to characterize hydrogen production in an AnMBR treating high-strength wastewater (COD > 1000 mg/L). Two aspects differentiate our model from existing literature: First, the model input is a multi-substrate wastewater that includes fractions of proteins, carbohydrates, and lipids. Second, the model integrates the ADM1 model with physical/biochemical processes that affect membrane performance (e.g., membrane fouling). The model includes mass balances of 27 variables in a transient state, where metabolites, extracellular polymeric substances, soluble microbial products, and surface membrane density were included. Model results showed the hydrogen production rate was higher when treating amino acids and sugar-rich influents, which is strongly related to higher EPS generation during the digestion of these metabolites. The highest H_2_ production rate for amino acid-rich influents was 6.1 LH_2_/L-d; for sugar-rich influents was 5.9 LH_2_/L-d; and for lipid-rich influents was 0.7 LH_2_/L-d. Modeled membrane fouling and backwashing cycles showed extreme behaviors for amino- and fatty-acid-rich substrates. Our model helps to identify operational constraints for H_2_ production in AnMBRs, providing a valuable tool for the design of fermentative/anaerobic MBR systems toward energy recovery.

## 1. Introduction

Among the existing technologies for biochemical waste-to-H_2_ production, including microbial fuel cells, microbial electrolysis cells, algae-catalyzed processes (biophotolysis and photofermentation), and even gas-separation MBR [1,2], those based on dark fermentation have the advantage of not requiring aeration or a light source for H_2_ production, facilitating their application in remote/decentralized areas where liquid wastes are available [3]. Many of these technologies, however, are still in their developmental stages, and urgent process intensification is required to cope with the growing demand for renewable hydrogen. Anaerobic membrane bioreactors (AnMBRs) are mature technologies traditionally used to decrease COD concentrations in high-strength waste streams [4,5]. However, this treatment objective has now switched to a more sustainable approach, where valuable resources such as nutrients, energy, and water can be recovered [6]. Depending on the reactor operation, AnMBRs can produce methane and/or hydrogen while generating high-quality effluents for further wastewater reclamation [7,8]. Several studies report their application for H_2_ recovery using non-competing feedstocks, such as food waste, agricultural residual waste (e.g., winery or sugar beet), animal-generated waste (e.g., dairy), organic fraction of municipal solid waste, or wastewater, among others [7,9,10,11,12,13]. However, information about biohydrogen production in AnMBRs is still limited to lab and pilot scales due to the stringent control of operational variables, the need for substrate pre-treatment, energy cost, membrane fouling, H_2_ stripping, OLR maintenance, or even microbial competition [14].

Modeling becomes an essential tool for understanding the behavior of complex systems like AnMBRs for H_2_ recovery [1]. A benchmark model to describe the biological stage in an AnMBR is the anaerobic digestion model 1 (ADM1) [15], developed for the digestion of high-strength wastewater (concentration of COD over 1000 mg/L in the influent) [16]. A modification to ADM1 was proposed by Siegrist et al. (2002) [17], where mesophilic and thermophilic conditions were studied during digestion. The main limitation of both models is that they were designed only to predict the biochemical activity inside a reactor. However, a complete model of AnMBR must include additional processes that account for the presence of the membrane unit. For instance, membrane fouling represents one of the highest costs in the operation and maintenance of AnMBRs. Due to high concentrations of organic matter, extracellular polymeric substances (EPS) and soluble microbial products (SMP) play a crucial role in membrane fouling [18,19]. Some authors have modeled the membrane fouling mechanisms in a submerged AnMBR in response to the SMP and EPS concentrations [20,21]. The critical limitation of these models is the use of single substrates (e.g., hexoses), which might not represent actual wastewater and could lead to idealistic results in hydrogen generation. Additionally, these models might not be extrapolated to more complex systems. Recent advances in AnMBR modeling include numerical and statistical techniques like machine/deep learning. However, reproducibility is problematic for these models since they are limited to the system where the data were collected [22,23,24]. A summary of the main model structures in the literature used for modeling AnMBR is shown in Table 1. Modeling structures often do not include biochemical and physical processes together, except those modeling membrane cake fouling due to EPS and SMP, which are limited to one substrate and focus on the EPS [21,25].

In this study, we developed a mechanistic model for hydrogen production in submerged AnMBR treating high-strength wastewater. The model builds on the ADM1 model and incorporates physical and biochemical processes to describe membrane fouling due to a multi-substrate influent (i.e., carbohydrates, proteins, and fats) and their impact on hydrogen production. Additionally, a sensitivity analysis established the key operating conditions of the system for H_2_ production. The study aims to provide a useful tool that accurately represents the physical and biochemical processes occurring in a submerged AnMBR treating a multi-substrate influent, to aid the design and simulation of the operation of this system for H_2_ recovery in high-strength waste streams. 

## 2. Materials and Methods

### 2.1. AnMBR Setup and Operational Conditions

**Figure 1:**
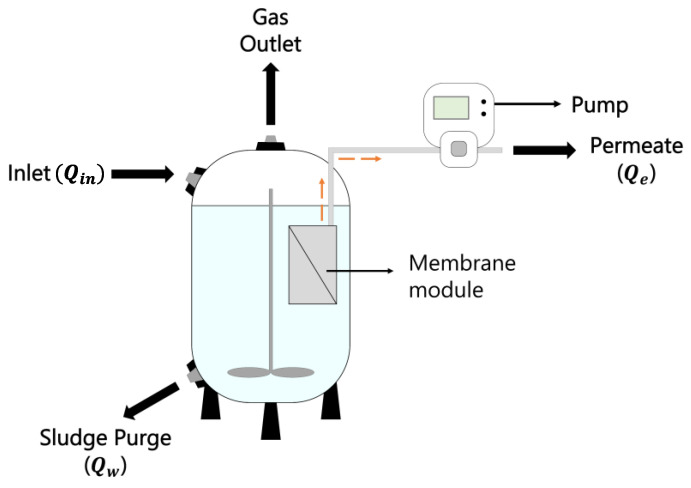
Schematic of the modeled system.

The modeled system consists of a continuous stirred tank reactor (CSRT) coupled to a submerged liquid-separation membrane. Figure 1 shows a schematic of the system, including the inlet flow (*Q_in_*), gas outlet, sludge purge (*Q_w_*), and permeate flow (*Q_e_*). Although the modeled processes were temperature- and pH-dependent, initial conditions were 35 °C and pH 7. Other parameters included inlet microbial concentration, COD concentration, and substrate composition (amino acids, sugars, long-chain fatty acids, and inert matter content). Initial values were 50 mg/L of microorganisms in the feed and a COD inlet of 4000 mg/L, as established by Siegrist et al., 2002 [17]. The reactor volume (*V*) was 1 m^3^ and the hydraulic retention time (HRT) was 12 h. For hydrogen production in AnMBRs, there is no standard value for solids retention time (SRT) in the current literature [29,30]. Thus, we selected a conservative SRT of 6 days as a starting point since some studies suggest SRT values higher than 15 days might decline H_2_ production rates [31]. Permeate flux was defined by $Q_{e}=V/HRT$, and the sludge purge flux was defined as $Q_{w}=V/SRT$. 

### 2.2. Modeling Hydrogen Production

The mass balances for the soluble compounds involved in the AnMBR model are shown in Equation (1).

(1) $$V\frac{dS_{i}}{dt}=Q_{in}S_{i}^{in}-Q_{e}S_{i}^{e}-Q_{w}S_{i}^{w}+r_{i}V$$

where *S_i_* corresponds to the concentration of a soluble specie *i*.

The mass balance for particulate compounds, microorganisms included, is shown in Equation (2). Complete retention by the membrane is assumed for particulate compounds.

(2) $$V\frac{dX_{i}}{dt}=Q_{in}X_{i}^{in}-Q_{w}X_{i}^{w}+r_{i}V$$

where *X_i_* corresponds to the concentration of a particulate specie *i*.

The rates of the processes (rows) involved in the consumption or generation of each compound inside the reactor (columns) are summarized in the Peterson Matrix (Table 2) and calculated with Equation (3).

(3) $$r_{i}=\overset{26}{\underset{i}{\sum }}\overset{20}{\underset{j}{\sum }}\nu_{j,i}\rho_{j}$$

where *r_i_* is the kinetic reaction rate law for a compound *i*, $\nu_{j,i}$ is a stoichiometric coefficient, and $\rho_{j}$ is the kinetic expression for a process *j*.

#### 2.2.1. Bioreactor Model Kinetics

The anaerobic digestion model includes the hydrolysis of particulate organic matter, fermentation and oxidation of metabolites, biomass growth and decay, and production and consumption of soluble microbial products (SMP) and extracellular polymeric substances (EPS). The processes involved in this model are described as follows.

- Degradation of particulate organic matter *ρ*_1_: Particulate matter is composed by macronutrients and dead biomass, which are hydrolyzed into amino acids, sugars, and long chain fatty acids (LCFA). This process is described in Equation (4).

(4) $$\rho_{1}=k_{H}X_{S}$$

where *k_H_* is the hydrolysis constant rate, and *X_S_* is the concentration of the total substrate.

- Fermentation of amino acids *ρ*_2_ and sugars *ρ*_3_: both processes were based on the Michaelis–Menten (MM) model (Equations (5) and (6)) and inhibited by pH.

(5) $$\rho_{2}=\mu_{max,2}\frac{S_{aa}}{K_{S,aa}+S_{aa}}I_{pH,2}X_{aa}$$

(6) $$\rho_{3}=\mu_{max,3}\frac{S_{su}}{K_{S,su}+S_{su}}I_{pH,2}X_{su}$$

where $\mu_{max,2}$ and $\mu_{max,3}$ are the maximum growth rates for fermentation, $S_{aa}$ and $S_{su}$ are the concentrations of amino acids and sugars, respectively; $K_{S,aa}$ and $K_{S,su}$ are the half-saturation constants; and $X_{aa}$ and *X_Su_* are the concentration of amino acids and sugar degraders.

- Anaerobic oxidation of LCFA *ρ*_4_: this process also follows a MM model; however, it presents inhibition due to acetate concentration, hydrogen concentration, and pH (Equation (7)).

(7) $$\rho_{4}=\mu_{max,4}\frac{S_{fa}}{K_{S,fa}+S_{fa}}I_{ac,4}I_{H_{2},4}I_{pH,4}X_{fa}$$

where $\mu_{max,4}$ is the maximum growth rate for anaerobic oxidation, $S_{fa}$ is the concentration of long chain fatty acids, $K_{s,fa}$ is the half-saturation constant for LCFA, and $X_{fa}$ is the concentration of LCFA degraders.

- Anaerobic oxidation of intermediary products *ρ*_5_: for propionate, the expression for oxidation is given by Equation (8), following the MM model. This process is inhibited by acetate, hydrogen, pH level and ammonia concentration.

(8) $$\rho_{5}=\mu_{max,5}\frac{S_{pro}}{K_{S,pro}+S_{pro}}I_{ac,5}I_{H_{2},5}I_{pH,6}I_{NH_{3}}X_{pro}\;$$

where $\mu_{max,5}$ is the maximum growth rate for oxidation, $S_{pro}$ is the concentration of propionate, $K_{S,fa}$ is the half-saturation constant for propionate, and $X_{pro}$ is the concentration of propionate degraders.

- Acetotrophic methanogenesis *ρ*_6_: based on the MM model and inhibited by pH level and ammonia concentrations.

(9) $$\rho_{6}=\mu_{max,6}\frac{S_{ac}}{K_{S,ac}+S_{ac}}I_{pH,6}I_{NH_{3}}X_{ac}$$

where $\mu_{max,6}$ is the maximum growth rate, $S_{ac}$ is the concentration of acetate, $K_{S,ac}$ is the half-saturation constant for acetate, and $X_{ac}$ is the concentration of acetate degraders.

- Hydrogenotrophic methanogenesis *ρ*_7_: based on the MM model and inhibited by ammonia and hydrogen concentrations (Equation (10)).

(10) $$\rho_{7}=\mu_{max,7}\frac{S_{H_{2}}}{K_{S,H_{2}}+S_{H_{2}}}I_{pH,6}I_{NH_{3}}X_{H_{2}}$$

where $\mu_{max,7}$ is the maximum growth rate, $S_{H_{2}}$ is the concentration of hydrogen, $K_{S,H_{2}}$ is the half-saturation constant for hydrogen, and $X_{H_{2}}$ is the concentration of hydrogen degraders.

- Biomass decay *ρ*_8_–*ρ*_13_: first order kinetics was assumed for decay (Equation (11)).

(11) $$\rho_{j}=k_{d,j}X_{i}$$

where $k_{d,j}$ is the kinetic decay constant, and $X_{i}$ is the concentration of a specific microorganism.

- Bicarbonate and dissolved carbon dioxide equilibrium *ρ*_14_: described in Equation (12), the kinetic expression is based on the equilibrium Equation (13).

(12) $$\rho_{14}=k_{eq,CO_{2}/HCO_{3}^{-}}\;\left(S_{HCO_{3}^{-}}S_{H^{+}}-S_{CO_{2}}K_{CO_{2}/HCO_{3}^{-}}\right)$$

(13) $$CO_{2}+H_{2}O↔HCO_{3}^{-}+H^{+}$$

where $k_{eq,CO_{2}/HCO_{3}^{-}}$ is the rate constant for carbon dioxide/carbonate equilibrium, $K_{CO_{2}/HCO_{3}^{-}}$ is the equilibrium constant for carbon dioxide/carbonate systema. $S_{HCO_{3}^{-}}$ is the concentration of the ion bicarbonate, $S_{H^{+}}$ is the concentration of protons, and $S_{CO_{2}}$ is the concentration of carbon dioxide.

- Ammonia and ammonium equilibrium *ρ*_15_: described in Equation (14), the kinetic expression is based on the equilibrium Equation (15).

(14) $$\rho_{15}=k_{eq,NH_{4}^{+}/NH_{3}}\;\left(S_{NH_{3}}S_{CO_{2}}-\frac{S_{NH_{4}^{+}}S_{HCO_{3}^{-}}K_{NH_{4}^{+}/NH_{3}}}{K_{CO_{2}/HCO_{3}^{-}}}\right)\;$$

(15) $$NH_{4}^{+}+HCO_{3}^{-}↔NH_{3}+CO_{2}+H_{2}O$$

where $k_{eq,NH_{4}^{+}/NH_{3}}$ is the rate constant for ammonia/ammonium equilibrium, $K_{NH_{4}^{+}/NH_{3}}$ is the equilibrium constant for ammonia/ammonium system, $S_{NH_{4}^{+}}$ is the concentration of ion ammonium, and $S_{NH_{3}}$ is the concentration of ammonia.

- Acetate and propionate protonation *ρ*_16_–*ρ*_17_: two pseudo equilibrium processes were considered (Equations (16) and (17)).

(16) $$\rho_{16}=k_{eq,hac/ac}\;\left(S_{ac}S_{CO_{2}}-\frac{S_{hac}S_{HCO_{3}^{-}}K_{hac/ac}}{K_{CO_{2}/HCO_{3}^{-}}}\right)\;$$

where $k_{eq,,hac/ac}$ is the rate constant for acetic acid/acetate equilibrium, $K_{hac/ac}$ is the equilibrium constant for acetic acid/acetate system, $S_{hac}$ is the concentration of the acetic acid, and $S_{ac}$ is the concentration of acetate.

(17) $$\rho_{17}=k_{eq,hpro/pro}\;\left(S_{ac}S_{CO_{2}}-\frac{S_{hpro}S_{HCO_{3}^{-}}K_{hpro/pro}}{K_{CO_{2}/HCO_{3}^{-}}}\right)\;$$

where $k_{eq,hpro/pro}$ is the rate constant for propionic acid/propionate equilibrium, $K_{hpro/pro}$ is the equilibrium constant for propionic acid/propionate system, $S_{hpro}$ is the concentration of the propionic acid, and $S_{pro}$ is the concentration of propionate.

- Inhibition processes: the following non-competitive inhibition expressions were considered.

(18) $$I_{ac,j}=\frac{K_{I,ac,j}}{K_{I,ac,j}+S_{ac}}$$

(19) $$I_{H_{2},j}=\frac{K_{I,H_{2},j}}{K_{I,H_{2},j}+S_{H_{2}}}$$

(20) $$I_{NH_{3},j}=\frac{K_{I,NH_{3},j}^{2}}{K_{I,NH_{3},j}^{2}+S_{NH_{3}}^{2}}$$

(21) $$I_{pH,j\;}=\frac{K_{I,NH_{3},j}^{2}}{K_{I,NH_{3},j}^{2}+S_{H^{+}}^{2}}$$

where $K_{I,ac,j}$, $K_{I,H_{2},j}$, $K_{I,NH_{3},j}^{2}$, and $K_{I,NH_{3},j}^{2}$ are the inhibition constants for acetate, hydrogen, ammonia, and pH, respectively; $S_{H_{2}}$ is the concentration of hydrogen, $S_{NH_{3}}$ is the concentration of ammonia, and $S_{H^{+}}$ is the concentration of protons.

- Temperature dependency: expressed by Equation (22).

(22) $$\gamma =\gamma_{35{}^{\circ}C}\cdot \exp\left(\theta \cdot \left(T-35\right)\right)$$

where $\gamma_{35{}^{\circ}C}$ is the value of a parameter at 35 °C, θ is the corrector parameter, and *T* is the objective temperature. Parameters with temperature dependency are shown in Table 3.

#### 2.2.2. Membrane Model Kinetics

The membrane model depends on biological processes that include the hydrolysis of EPSs to BAPs, formation of BAPs and UAPs in proportion to the substrate utilization, and biodegradation of BAPs and UAPs. The formation of BAPs, UAPs, and EPSs from a multi-substrate is one of the main attributes of the current study since these processes are often modeled to consider a single substrate. The model does not account for membrane sparging, pH, temperature control, and fluid dynamics inside the tank. The kinetic parameters related to the mentioned processes are described as follows.

- BAP and UAP decay *ρ*_18_–*ρ*_19_: these processes were modeled following the expression developed by Jang et al., 2006 [20], which established MM mechanisms for the decay, as shown in Equations (23) and (24).

(23) $$\rho_{18}=k_{d,BAP}\frac{S_{BAP}}{K_{S,BAP}+S_{BAP}}X_{a}$$

(24) $$\rho_{19}=k_{d,UAP}\frac{S_{UAP}}{K_{S,UAP}+S_{UAP}}X_{a}$$

where $k_{d,BAP}$ and $k_{d,UAP}$ are the maximum specific substrate utilization rates for BAP and UAP, $K_{S,BAP}$ and $K_{S,UAP}$ are the half-saturation constants for BAP and UAP, $S_{BAP}$ and $S_{UAP}$ are the BAP and UAP concentration, and $X_{a}$ is the active biomass $\left(\sum_{i}^{n}X_{i}\right)$.

- EPS decay *ρ*_20_: first order kinetics was assumed for this process (Equation (25)).

(25) $$\rho_{20}=k_{2}S_{EPS}$$

where *k*_2_ is the BAP formation rate coefficient, and $S_{EPS}$ is the concentration of EPS.

- Fouling model: The accumulation of EPS density on the membrane surface (*m*) can be expressed as shown in Equation (26).

(26) $$\frac{dm}{dt}=JS_{EPS}-k_{dm}m$$

where *J* is the flux through the membrane, and $k_{dm}$ is the detachment rate of the EPS from the membrane (Equation (27)).

(27) $$k_{dm}=\eta \left(\tau_{m}-\Delta_{m}\Delta P\right)$$

where η is a constant, $\tau_{m}$ is the shear stress, Δ*_m_* is the static friction coefficient, and Δ*P* is the transmembrane pressure. In addition, the flux can be expressed as

(28) $$J=\frac{\Delta P}{\mu \left(\alpha_{s}m+R_{m}\right)}$$

where μ is the dynamic viscosity of the permeate, $\alpha_{s}$ is the specific resistance of EPS, and $R_{m}$ is the membrane resistance.

Finally, backwashing frequency (BW) was set according to Yoon (2005) [32] for a membrane filtration performance under recommended operational conditions (transmembrane pressure should not exceed 30 kPa).

#### 2.2.3. Liquid–Gas Mass Transfer

Mass transfer from the liquid to the gas phase was modeled according to Equation (29).

(29) $$F_{j}=-k_{j}\left(S_{j,interface}-S_{j}\right)$$

where $k_{j}$ is the mass transfer coefficient for analyte *j*, $S_{j,interface}$ is the concentration of j in the interface, and $S_{j}$ is the concentration of the analyte *j* in the liquid bulk.

$S_{j,interface}$ was estimated according to Equation (30).

(30) $$S_{j,interface}=\frac{p_{j}}{H_{j}\exp\left(\theta_{Henry}T\right)}$$

where $p_{j}$ is the partial pressure of j in the gas section, $H_{j}$ is the Henry’s constant for *j*, $\theta_{Henry}$ is a temperature correction factor, and *T* is the operation temperature. Partial pressure $p_{j}$ was estimated using the ideal gases law.

**Table 2:** Peterson matrix of interactions.

Units	mol-m^3^	mgCOD-m^3^	g-m^3^	mol-m^3^	mol-m^3^	g-m^3^	g-m^3^	gCOD-m^3^	gCOD-m^3^	gCOD-m^3^	gCOD-m^3^	gCOD-m^3^	gCOD-m^3^
n° component	1	2	3	4	5	6	7	8	9	10	11	12	13
Process	$S_{H^{+}}$	$S_{H_{2}}$	$S_{CH_{4}}$	$S_{CO_{2}}$	$S_{HCO_{3}^{-}}$	$S_{NH_{4}^{+}}$	$S_{NH_{3}}$	$S_{ac}$	$S_{hac}$	$S_{pro}$	$S_{hpro}$	$S_{aa}$	$S_{su}$
*ρ*_1_				0.0004	−0.0005							0.30	0.2
*ρ*_2_		0.96		0.043	−0.022	0.587		3.29		1.42		−6.67	
*ρ*_3_		0.96		0.091	−0.07	−0.08		3.29		1.42			−6.67
*ρ*_4_		6.70		0.199	−0.202	−0.08		14.3					
*ρ*_5_		8.20		0.162	0.004	−0.08		10.8		−20			
*ρ*_6_			39.0	−0.006	0.618	−0.08		−40.0					
*ρ*_7_		−22.0	21.0	−0.353	−0.006	−0.08							
*ρ*_8_					0.003	0.045							
*ρ*_9_					0.003	0.045							
*ρ*_10_					0.003	0.045							
*ρ*_11_					0.003	0.045							
*ρ*_12_					0.003	0.045							
*ρ*_13_					0.003	0.045							
*ρ*_14_	−1			1	−1								
*ρ*_15_				−1	1	14.0	−14.0						
*ρ*_16_				−1	1			−64.0	64				
*ρ*_17_				−1	1					−112	112		
*ρ*_18_													
*ρ*_19_													
*ρ*_20_													
n° component	14	15	16	17	18	19	20	21	22	23	24	25	26
Process	$S_{fa}$	$S_{in}$	$X_{S}$	$X_{aa}$	$X_{su}$	$X_{fa}$	$X_{pro}$	$X_{ac}$	$X_{H_{2}}$	$X_{in}$	$S_{BAP}$	$S_{UAP}$	$S_{EPS}$
*ρ*_1_	0.45	0.05	−1										
*ρ*_2_				1-*k_EPS_*-*k*_1_								*k*_1_	*k_EPS_*
*ρ*_3_					1-*k_EPS_*-*k*_1_							*k*_1_	*k_EPS_*
*ρ*_4_	−22.0					1-*k_EPS_*-*k*_1_						*k*_1_	*k_EPS_*
*ρ*_5_							1-*k_EPS_*-*k*_1_					*k*_1_	*k_EPS_*
*ρ*_6_								1-*k_EPS_*-*k*_1_				*k*_1_	*k_EPS_*
*ρ*_7_									1-*k_EPS_*-*k*_1_			*k*_1_	*k_EPS_*
*ρ*_8_			0.8	−1						0.2			
*ρ*_9_			0.8		−1					0.2			
*ρ*_10_			0.8			−1				0.2			
*ρ*_11_			0.8				−1			0.2			
*ρ*_12_			0.8					−1		0.2			
*ρ*_13_			0.8						−1	0.2			
*ρ*_14_													
*ρ*_15_													
*ρ*_16_													
*ρ*_17_													
*ρ*_18_				$Y_{p}f_{aa}$	$Y_{p}f_{su}$	$Y_{p}f_{fa}$	$Y_{p}f_{pro}$	$Y_{p}f_{ac}$	$Y_{p}f_{h2}$		−1		
*ρ*_19_				$Y_{p}f_{aa}$	$Y_{p}f_{su}$	$Y_{p}f_{fa}$	$Y_{p}f_{pro}$	$Y_{p}f_{ac}$	$Y_{p}f_{h2}$			−1	
*ρ*_20_											1		−1

### 2.3. Model Parameters and Numerical Techniques

The parameters used for the model solution are summarized in Table 3. For this study, a transient state for a CSTR was assumed. The developed model was solved using ode15s with non-negative condition from MATLAB (The MathWorks Inc., Natick, MA, USA).

To check for the stability of the model, the model’s steady state as a function of the initial conditions was evaluated. The model was set to an inlet total substrate concentration of 10,000 $\frac{mg_{COD}}{L}$, and the following initial conditions: (a) MLSS > 0, $COD_{initial}$ = 0 $\frac{mg_{COD}}{L}$; (b) MLSS = 0, $COD_{initial}$= 0 $\frac{mg_{COD}}{L}$; (c) MLSS > 0, $COD_{initial}$ = 5000$\frac{mg_{COD}}{L}$; (d) MLSS > 0, $COD_{initial}$ = 10,000 $\frac{mg_{COD}}{L}$.

### 2.4. Model Response and Sensitivity Analysis

To analyze the response of the model to changes in the inlet concentration and composition, we evaluated influent/inlet configurations presented in Table 4. Additionally, we evaluated the same inlet configurations, along with variable backwashing protocols, to observe the response of the EPS membrane surface density and the transmembrane pressure (TMP).

For the sensitivity analysis, critical parameters affecting the hydrogen production were determined by using the one-factor-at-a-time (OAT) technique. We varied the kinetic and the operational parameters (SRT, HRT, and temperature) by ±50% to identify their impact on the hydrogen production.

**Table 3:** Parameters used in the model.

Parameter	Value	Units	θ (°C^−1^)	Reference
$k_{H}$	0.25	*d* ^−1^	0.024	[17]
$\mu_{max,2}$	4	*d* ^−1^	0.069	
$\mu_{max,3}$	4	*d* ^−1^	0.069	
$\mu_{max,4}$	0.6	*d* ^−1^	0.055	
$\mu_{max,5}$	0.6	*d* ^−1^	0.055	
$\mu_{max,6}$	0.37	*d* ^−1^	0.069	
$\mu_{max,7}$	2	*d* ^−1^	0.069	
$k_{d,8}$	0.8	*d* ^−1^	0.069	
$k_{d,9}$	0.8	*d* ^−1^	0.069	
$k_{d,10}$	0.06	*d* ^−1^	0.055	
$k_{d,11}$	0.06	*d* ^−1^	0.055	
$k_{d,12}$	0.05	*d* ^−1^	0.069	
$k_{d,13}$	0.3	*d* ^−1^	0.069	
$k_{d,BAP}$	0.07	$\frac{mg_{BAP}}{mg_{X_{a}}-d}$	-	[20]
$k_{d,UAP}$	0.4	$\frac{mg_{UAP}}{mg_{X_{a}}-d}$	-	
$K_{S,aa}$	50	$\frac{mg}{L}$	0.069	[17]
$K_{S,su}$	50	$\frac{mg}{L}$	0.069	
$K_{S,fa}$	1000	$\frac{mg}{L}$	0.035	
$K_{S,pro}$	20	$\frac{mg}{L}$	0.10	
$K_{S,ac}$	40	$\frac{mg}{L}$	0.10	
$K_{S,h2}$	1	$\frac{mg}{L}$	0.08	
$K_{S,BAP}$	85	$\frac{mg}{L}$	-	[20]
$K_{S,UAP}$	100	$\frac{mg}{L}$	-	
$k_{eqCO_{2}/HCO_{3}^{-}}$	10	$\frac{m^{3}}{mol-d}$	-	[17]
$k_{eqNH_{4}^{+}/NH_{3}}$	10	$\frac{m^{3}}{g-d}$	-	
$k_{eqhac/ac}$	10	$\frac{m^{3}}{g-d}$	−0.004	
$k_{eqhpro/pro}$	10	$\frac{m^{3}}{g-d}$	−0.004	
$K_{CO_{2}/HCO_{3}^{-}}$	$7.1\cdot 10^{-4}$	$\frac{mol}{m^{3}}$	0.004	
$K_{NH_{4}^{+}/NH_{3}}$	$10^{-6}$	$\frac{mol}{m^{3}}$	0.063	
$K_{hac/ac}$	0.025	$\frac{mol}{m^{3}}$	-	
$K_{hpro/pro}$	0.019	$\frac{mol}{m^{3}}$	-	
$K_{I,ac,4-5}$	1500	$\frac{mg}{L}$	-	
$K_{I,H_{2},4}$	3	$\frac{\mu g}{L}$	0.08	
$K_{I,H_{2},5}$	1	$\frac{\mu g}{L}$	0.08	
$K_{I,pH,2-3}$	0.01	$\frac{mol}{m^{3}}$	-	
$K_{I,pH,4-7}$	$5\cdot 10^{-4}$	$\frac{mol}{m^{3}}$	-	
$K_{I,NH_{3},5}$	25	$\frac{mg}{L}$	0.061	
$K_{I,NH_{3},6}$	17	$\frac{mg}{L}$	0.086	
$k_{1}$	0.05	$\frac{mg_{UAP}}{mg_{S}}$	-	[20]
$k_{2}$	0.02	$\frac{mg_{BAP}}{mg_{EPS}-d}$	-	
η	0.1	$\frac{1}{Pa-d}$	-	[25]
$\tau_{m}$	5	Pa	-	[33]
$\Delta_{m}$	$10^{-3}$	-	-	[25]
μ	0.0013	Pa−s	-	[21]
$a_{s}$	$5\cdot 10^{12}$	$\frac{m}{kg}$	-	
$R_{m}$	$1.45\cdot 10^{12}$	*m* ^−1^	-	
$H_{H_{2}}$	58	-	−0.002	[17]
$H_{CO_{2}}$	1.65	-	0.017	

**Table 4:** Inlet variable composition to evaluate model response.

Case	COD (mg/L)	%Amino Acids	%Sugars	%Fatty Acids	%Inert Matter
A	2000	30	20	45	5
B	4000				
C	7000				
D	10,000				
E	20,000				
1	10,000	100	0	0	0
2		0	100	0	0
3		0	0	100	0
4		30	20	45	5
5		30	45	20	5
6		31.3	46.3	21.3	0
7		30	45	20	5
8		31.66	31.66	31.66	5
9		31.66	21.66	46.66	0

## 3. Results and Discussion

### 3.1. Steady State Analysis

**Figure 2:**
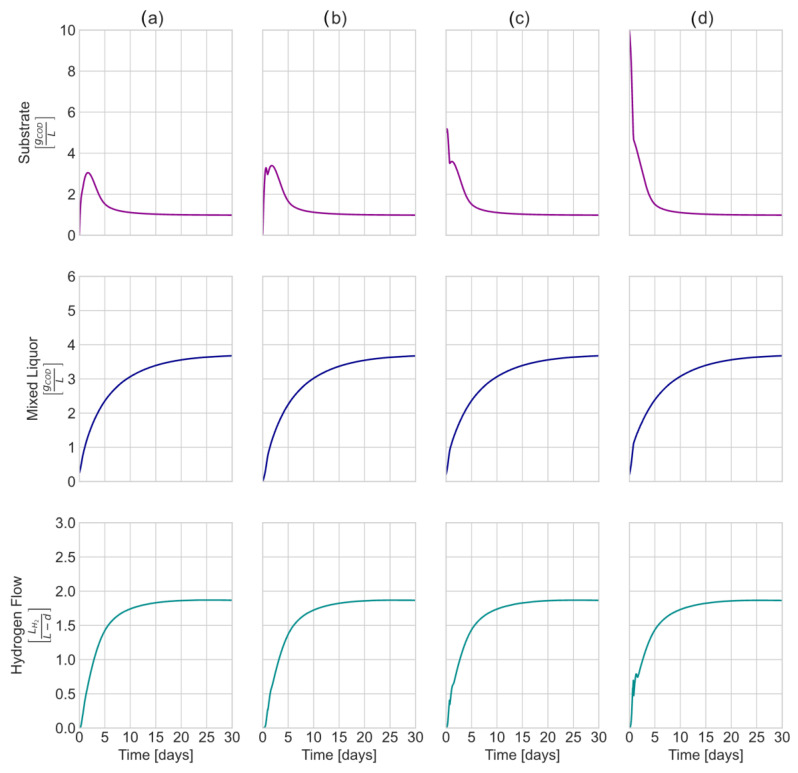
Evaluation of different steady states due to changes in initial conditions. For all four simulations, CODinlet = 10,000 mgCOD/L. (**a**) Biomass inside the reactor > 0, CODinitial = 0. (**b**) Biomass inside the reactor = 0, CODinitial = 0. (**c**) Biomass inside the reactor > 0, CODinitial = 5000 mgCOD/L. (**d**) Biomass inside the reactor > 0, CODinitial = 10,000 mgCOD/L.

Figure 2 shows the results of different paths to the steady state from different initial conditions. The simulation indicates that the steady state for the substrate, biomass concentration, and hydrogen flow remains constant for all the tested conditions. At low COD initial concentrations, the curves remain smooth. However, once the reactor is fed (transient state), noise begins to appear in the curves as the concentration increases (Figure 2c,d). The explanation for this phenomenon lies in the expressions of generation and consumption of each of the metabolites. The algebraic expressions for generation and/or consumption directly depend on the metabolites’ concentration. As a result, irregularities in the curves could be due to high derivative values. This behavior is typical for ADM1 and ADM1-based models, as an overprediction of the metabolites’ concentrations is often reported under start-up conditions [17]. For design and scale-up purposes, it is essential to consider steady-state conditions.

### 3.2. AnMBR Model Behavior at Variable CODinlet and Substrate Composition

**Figure 3:**
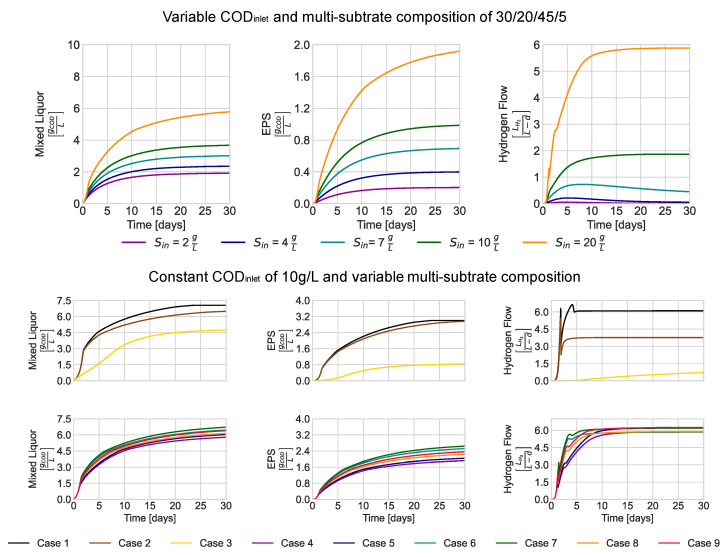
Modeled biomass, EPS, and hydrogen output for variable COD and multi-substrate composition (amino-acids/sugars/fatty-acids/inert-matter). Case 1—100/0/0/0, Case 2—0/100/0/0, Case 3—0/0/100/0, Case 4—30/20/45/5, Case 5—30/45/20/5, Case 6—31.3/46.3/21.3/0, Case 7—30/45/20/5, Case 8—31.66/31.66/31.66/5, Case 9—31.66/21.66/46.66/0.

Figure 3 illustrates the simulation results for the evolution of biomass, EPS, and hydrogen, considering different values of inlet COD with a multi-substrate composition of 30% amino acids, 20% sugars, 45% fatty acids, and 5% inert matter [17]. The chosen COD values are representative of high-strength wastewater, as described by Shin et al. (2021) [16]. The simulation exhibited the expected behavior for each set of CODinlet concentrations, showing increasing biomass, EPS, and hydrogen production with higher CODinlet. However, the biogas composition depended on the substrate composition. Specifically, when considering a 10 g/L CODinlet with variable content of amino acids/sugars/fatty acids/inert matter, the largest hydrogen production was observed with a 100% amino acids substrate (Case 1). However, this substrate composition also generated the highest EPS concentrations in the mixed liquor, potentially impacting membrane durability and performance [34,35]

Stoichiometrically, hydrogen production should be higher with a 100% sugar substrate (Case 2). However, the results indicated possible inhibition in H_2_ production from propionate oxidation due to carbon dioxide accumulation (Equation (17)). When considering a hypothetical waste stream composed solely of fatty acids, biomass growth was lower compared to those with 100% content of amino acids or sugars (Cases 1 and 2). Hydrogen production and EPS were also limited, mainly because fatty acids were not involved in propionate generation, one of the start-up metabolites in the modeled hydrogen production. Additionally, LCFA kinetics were slower compared to sugars and amino acids. Overall, the results of the simulations suggest an enhanced hydrogen production in the AnMBR when treating multi-substrate influents rather than single-substrate ones.

**Figure 4:**
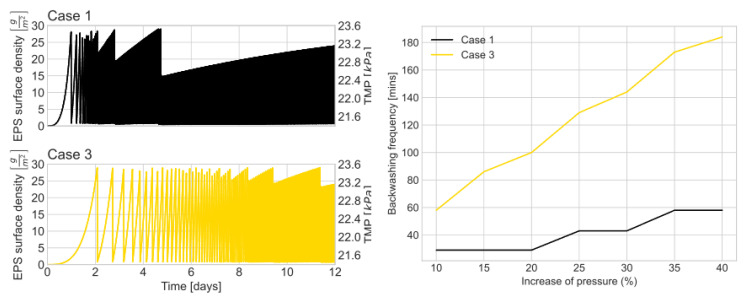
Left: Effect of inlet composition in EPS surface density and TMP. Time series follows the same trend for both EPS and TMP. Right: Effect of increasing transmembrane pressure tolerance in backwashing frequency. Case 1—100/0/0/0, Case 3—0/0/100/0.

**Figure 5:**
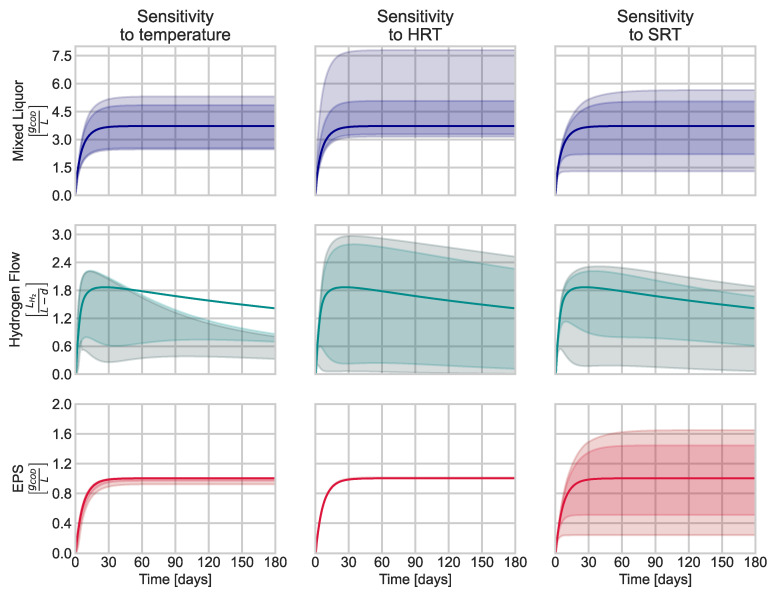
Changes in concentration of MLSS, biohydrogen production rate, and EPS concentration in relation to temperature, HRT, SRT. This simulation was obtained for HRT of 12 h, SRT of 6 d, inlet COD of 10 g/L, and a temperature of 35 °C. The figure shows an OAT analysis for variations of 50% (darkest) and 70% (lighter) for the mentioned design variables.

Regarding membrane operation, Figure 4 illustrates the fouling control cycles for cases 1 and 3. Backwashing occurs when the transmembrane pressure reaches a value 10% higher than the initial pressure (m = 0, Equation (20)). This restriction leads to an EPS surface density close to 30 g/m^2^. Figure 5 focuses on cases 1 and 3, representing the extremes of all the simulated cases. Influent with higher fatty acid concentrations results in less EPS production and, consequently, less frequent backwashing. Conversely, influents with a higher amino acid content (Figure 4, Case 1) require more membrane fouling control, leading to more frequent backwashing. To further analyze the data, Table 5 summarizes the number of events and backwashing frequency for different influent compositions. The number of backwashing events for all the evaluated cases tends to stabilize after four SRTs or 24 days from the start of operation once the EPS concentration reaches a steady state. Generally, influents with higher content of sugars and amino acids require more frequent backwashing events due to their strong relationship with EPS generation.

It is important to highlight that the backwashing frequency also changes as a function of the transmembrane pressure restrictions (Figure 4). As expected, backwashing is less frequent when the transmembrane pressure tolerance is higher. Since there is not an optimal value set in the current literature, then the condition for backwashing must be set according to the user’s design and operational criteria (e.g., membrane lifetime, energy efficiency, etc.) [36,37]. When TMP is allowed to increase, the membrane is overstressed, which could reduce its lifespan [38]. On the contrary, more frequent backwashing cycles might extend the membrane’s life, resulting in higher energy demand due to pumping.

### 3.3. Sensitivity Analysis

**Figure 6:**
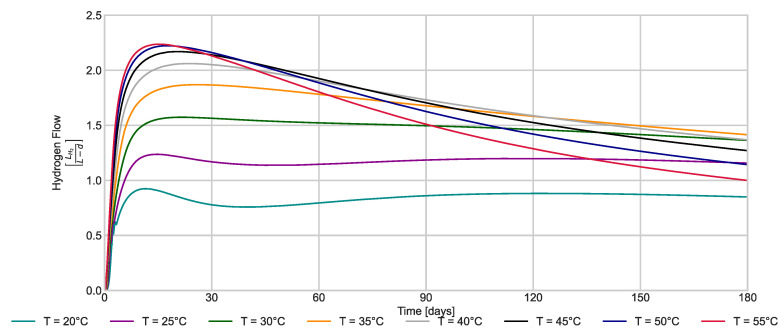
Hydrogen rate production at variable temperatures.

Figure 5 presents the results of the OAT analysis for three variables that notably impact hydrogen production and membrane fouling. Temperature changes (±50% change) directly affect the kinetic expressions governing mixed liquor solids (i.e., biomass) and hydrogen flow. The OAT analysis reveals that the temperature should be maintained under mesophilic conditions to increase hydrogen production, as there is no significant advantage observed with thermophilic operation. Higher temperatures increase hydrogen solubility, promoting inhibition (Equations (18)–(21)). Even though the model suggests less hydrogen production at room temperature, the hydrogen saturation is slower but remains constant (Figure 6). These results are consistent with existing laboratory studies that report higher bioH_2_ production at ambient or mesophilic temperatures than those reported for thermophilic reactors [39]. In addition, the microbial ecology is more diverse in reactors under mesophilic conditions [40]. Finally, increasing the reactor temperature is directly associated with higher energy consumption and costs.

Other parameters also have an important effect on the mixed liquor solid concentration. For instance, increasing HRT provides more contact time for substrate degradation and biomass growth. However, HRT also determines the membrane flux, which determines the TMP. Only a few laboratory studies report better H_2_ yields for HRTs between 8 and 9 h in AnMBRs [41,42]. Thus, finding the ideal HRT for hydrogen production in AnMBRs is a critical aspect that requires further research and consideration. Other factors such as substrate composition, influent COD, and operational conditions also play a role in determining the optimal HRT for efficient hydrogen production in AnMBRs. Therefore, a comprehensive approach is necessary to determine the most suitable operating conditions for maximizing hydrogen production while maintaining membrane performance and minimizing fouling.

The OAT shows that the SRT is a key parameter for bioH_2_ production and membrane fouling in an AnMBR. Older biomass might be detrimental to bioH_2_ production. Longer SRT reduces the sludge purge, which allows a more concentrated mixed liquor. Higher concentrations of mixed liquor solids increase hydrogen production, affecting EPS concentration and membrane backwashing frequency (Figure 4 and Table 5).

### 3.4. Model Results for H_2_ Production

Table 6 shows a comparison between the results of this model and the reported systems for hydrogen production. The existing literature suggests higher hydrogen production rates between 2.5 and 5.8 for submerged AnMBRs using sugar monomers as substrate. Some authors report improved H_2_ production in submerged AnMBR by up to 51% compared to the CSTR without a membrane [43]. By providing additional resistance to the permeate flow, the membrane can act as a degassing mechanism in an AnMBR [7]. However, only a few studies report H_2_ productivity while treating complex or multi-substrate effluents. For instance, Lee et al. (2014) [44] reported an H_2_ production rate of 10.7 while treating food waste with an inlet COD of 52.7 g/L. Although our study’s resulting H_2_ production rates are within the production ranges in the existing literature, more information about the substrate composition is required for further validation using reported data. Nevertheless, the developed model in this study serves as a helpful tool to identify operational constraints for H_2_ production in AnMBRs.

## 4. Conclusions

We developed a mechanistic model for hydrogen production in a submerged AnMBR. Two aspects differentiate our model from existing literature: First, the model input is a multi-substrate wastewater that includes fractions of proteins, carbohydrates, and lipids. Second, the model integrates the ADM1 model with physical/biochemical processes that affect membrane performance (e.g., membrane fouling). The simulated hydrogen production rates for multi-substrates showed better results than those for mono-substrates (e.g., glucose), specifically when treating amino acids and sugar-rich influents. The highest H_2_ production rate for amino acid-rich influents was 6.1 LH_2_/L-d; for sugar-rich influents was 5.9 LH_2_/L-d; and for lipid-rich influents was 0.7 LH_2_/L-d. Modeled membrane fouling and backwashing cycles showed extreme behaviors for amino-acid- and fatty-acid-rich substrates. Finally, mesophilic operation shows promising results for sustaining long-term H_2_ production in AnMBR.

The developed model is a valuable tool for the process intensification of H_2_ production using fermentative/anaerobic MBR systems; however, further research should include model validation using experimental data. In particular, data from AnMBRs treating multi-substrate effluents are required to optimize the operational conditions for H_2_ production.

## Figures and Tables

**Table 1:** Summary of modeling structures for AnMBR in the literature. These include reactor configuration, biochemical and physical processes, and treatment objectives.

Model	Reactor Type	Biochemical Processes	Membrane Processes	Objective	Source
ADM 1	CSTR	Hydrolysis of carbohydrates, proteins, lipids. Uptake of sugars, amino acids, LCFA, butyrate, propionate, acetate, and hydrogen. Growth and decay of microorganisms	NA	Describe the anaerobic digestion, quantifying the degradation and consumption of macronutrients, monomers, gases, and biomass.	[15]
First order dynamic model	Not specific	Degradation of VS	NA	To be an easy tool to predict biogas generation.	[26]
Modified Gompertz model	Batch biogas reactor	Production of biogas	NA	Describe biogas generation from a non-linear regression obtained from empirical observations.	[27]
Artificial Neural Networks	Not specific	Not specific	Not specific	Predict the behavior of systems based on collected empiric data from them.	[26,28]
Membrane cake fouling model due to EPS	SAnMBR	Substrate degradation. Growth and decay of microorganism. Production of EPS.	Membrane fouling. Transmembrane pressure.	Elucidate the membrane fouling due to EPS in SAnMBR and its impact in membrane durability.	[21,25]

**Table 5:** Effect of inlet composition in the backwashing frequency (HRT 12 h).

SRT (6 d)	No of SRTs Since Start of Operation					Min. Average Frequency	Max. Average Frequency
	1	2	3	4	5		
Case	(0–6) d	(6–12) d	(12–18) d	(18–24) d	(24–30) d	Min	Min
1	160	301	301	301	301	54	29
2	153	301	301	301	301	56	29
3	16	85	128	150	150	540	58
4	107	224	300	300	300	81	29
5	113	242	300	300	300	76	29
6	134	292	301	301	301	64	29
7	137	300	300	300	300	63	29
8	125	271	301	301	301	69	29
9	128	281	300	300	300	68	29

**Table 6:** Comparison among different operational systems for hydrogen production and this study.

Reactor Configuration	Substrate	Inlet COD (gCOD/L)	OLR (kg/m^3^-d)	HRT (h)	SRT (d)	TMP (kPa)	Productivity (L H_2_/L-d)	Reference
External loop	Glucose	10	68.0–92.7	3.3–5	2	14	9.2	[45]
External loop	3 Hexoses	20	120.0–480.0	1–4	Unknown	Unknown	66	[31]
Submerged	Glucose	10	26.7	9	450	70	2.5	[46]
Submerged	Glucose	10	40.0	8	1	Unkwown	4.5	[41]
Submerged	Glucose	17	37.5–44.3	9	2–90	Unknown	5.8	[42]
Submerged	Food waste	52.7	100.2	14	5.37	Unknown	10.7	[44]
Submerged *	Case 1 (protein rich)	10	21.7	12	6	21–23	6.1	This study
	Case 2 (sugar rich)						3.8	
	Case 3 (fat rich)						0.7	
	Case 4						5.9	
	Case 5						6.2	
	Case 6						5.8	
	Case 7						6.2	
	Case 8						5.9	
	Case 9						6.2	
CSTR **	Tofu processing waste	6.3	18.9	8	-	-	8.17	[47]
CSTR **	Cheese whey	60.5	242.0	6	-	-	2.9	[48]
CSTR **	Lactose	20	80.0	6	-	-	2.0	[49]

* Content of amino-acids/sugars/fatty-acids/inert-matter composition. Case 1—100/0/0/0, Case 2—0/100/0/0, Case 3—0/0/100/0, Case 4—30/20/45/5, Case 5—30/45/20/5, Case 6—31.3/46.3/21.3/0, Case 7—30/45/20/5, Case 8—31.66/31.66/31.66/5, Case 9—31.66/21.66/46.66/0. ** Studies for H_2_ production with raw wastes. No membrane applied.

## References

[1] Boese-Cortés I., Díaz-Alvarado F.A., Prieto A.L. (2023). Biocatalytic membrane reactor modeling for fermentative hydrogen production from wastewater: A review. Int. J. Hydrogen Energy.

[2] Goveas L.C., Nayak S., Kumar P.S., Vinayagam R., Selvaraj R., Rangasamy G. (2023). Recent advances in fermentative biohydrogen production. Int. J. Hydrogen Energy..

[3] Aziz M., Darmawan A., Juangsa F.B. (2021). Hydrogen production from biomasses and wastes: A technological review. Int. J. Hydrogen Energy.

[4] Yang W., Cicek N., Ilg J. (2006). State-of-the-art of membrane bioreactors: Worldwide research and commercial applications in North America. J. Membr. Sci..

[5] Wang Z., Wu Z., Mai S., Yang C., Wang X., An Y., Zhou Z. (2008). Research and applications of membrane bioreactors in China: Progress and prospect. Sep. Purif. Technol..

[6] Zielińska M., Ojo A. (2023). Anaerobic membrane bioreactors (ANMBRs) for wastewater treatment: Recovery of nutrients and energy, and management of fouling. Energies.

[7] Prieto A.L., Sigtermans L.H., Mutlu B.R., Aksan A., Arnold W.A., Novak P.J. (2016). Performance of a composite bioactive membrane for H_2_production and capture from high strength wastewater. Environ. Sci. Water Res. Technol..

[8] Kong Z., Li L., Wu J., Wang T., Rong C., Luo Z., Pan Y., Li D., Li Y., Huang Y. (2021). Evaluation of bio-energy recovery from the anaerobic treatment of municipal wastewater by a pilot-scale submerged anaerobic membrane bioreactor (AnMBR) at ambient temperature. Bioresour. Technol..

[9] Zhang M.-L., Fan Y.-T., Xing Y., Pan C.-M., Zhang G.-S., Lay J.-J. (2007). Enhanced biohydrogen production from cornstalk wastes with acidification pretreatment by mixed anaerobic cultures. Biomass-Bioenergy.

[10] Karlsson A., Vallin L., Ejlertsson J. (2008). Effects of temperature, hydraulic retention time and hydrogen extraction rate on hydrogen production from the fermentation of food industry residues and manure. Int. J. Hydrogen Energy.

[11] Ren N., Wang A., Cao G., Xu J., Gao L. (2009). Bioconversion of lignocellulosic biomass to hydrogen: Potential and challenges. Biotechnol. Adv..

[12] Ahmad T., Aadil R.M., Ahmed H., Rahman U.U., Soares B.C., Souza S.L., Pimentel T.C., Scudino H., Guimarães J.T., Esmerino E.A. (2019). Treatment and utilization of dairy industrial waste: A review. Trends Food Sci. Technol..

[13] Panigrahi S., Dubey B.K. (2019). A critical review on operating parameters and strategies to improve the biogas yield from anaerobic digestion of organic fraction of municipal solid waste. Renew. Energy.

[14] Banu J.R., Usman T.M., Kavitha S., Yukesh Kannah R., Yogalakshmi K.N., Sivashanmugam P., Bhatnagar A., Kumar G. (2021). A critical review on limitations and enhancement strategies associated with biohydrogen production. Int. J. Hydrogen Energy.

[15] Batstone D.J., Keller J., Angelidaki I., Kalyuzhnyi S.V., Pavlostathis S.G., Rozzi A., Sanders W.T.M., Siegrist H.A., Vavilin V.A. (2002). The IWA Anaerobic Digestion Model No 1 (ADM1). Water Sci. Technol..

[16] Shin C., Tilmans S.H., Chen F., Criddle C.S. (2021). Anaerobic membrane bioreactor model for design and prediction of domestic wastewater treatment process performance. Chem. Eng. J..

[17] Siegrist H., Vogt D., Garcia-Heras J.L., Gujer W. (2002). Mathematical model for meso- and thermophilic anaerobic sewage sludge digestion. Environ. Sci. Technol..

[18] Chen R., Nie Y., Hu Y., Miao R., Utashiro T., Li Q., Xu M., Li Y.-Y. (2017). Fouling behaviour of soluble microbial products and extracellular polymeric substances in a submerged anaerobic membrane bioreactor treating low-strength wastewater at room temperature. J. Membr. Sci..

[19] Maaz M., Yasin M., Aslam M., Kumar G., Atabani A., Idrees M., Anjum F., Jamil F., Ahmad R., Khan A.L. (2019). Anaerobic membrane bioreactors for wastewater treatment: Novel configurations, fouling control and energy considerations. Bioresour. Technol..

[20] Jang N., Ren X., Cho J., Kim I.S. (2006). Steady-state modeling of bio-fouling potentials with respect to the biological kinetics in the submerged membrane bioreactor (SMBR). J. Membr. Sci..

[21] Gautam R.K., Kamilya T., Verma S., Muthukumaran S., Jegatheesan V., Navaratna D. (2022). Evaluation of membrane cake fouling mechanism to estimate design parameters of a submerged AnMBR treating high strength industrial wastewater. J. Environ. Manag..

[22] Hosseinzadeh A., Zhou J.L., Altaee A., Li D. (2022). Machine learning modeling and analysis of biohydrogen production from wastewater by dark fermentation process. Bioresour. Technol..

[23] Li G., Ji J., Ni J., Wang S., Guo Y., Hu Y., Liu S., Huang S.-F., Li Y.-Y. (2022). Application of deep learning for predicting the treatment performance of real municipal wastewater based on one-year operation of two anaerobic membrane bioreactors. Sci. Total Environ..

[24] Yao J., Wu Z., Liu Y., Zheng X., Zhang H., Dong R., Qiao W. (2022). Predicting membrane fouling in a high solid AnMBR treating OFMSW leachate through a genetic algorithm and the optimization of a BP neural network model. J. Environ. Manag..

[25] Nagaoka S.H., Ueda A.M. (1996). Influence of bacterial extracellular polymers on the membrane separation activated sludge process. Water Sci. Technol..

[26] Kunatsa T., Xia X. (2022). A review on anaerobic digestion with focus on the role of biomass co-digestion, modelling and optimisation on biogas production and enhancement. Bioresour. Technol..

[27] Etuwe C.N., Momoh Y.O.L., Iyagba E.T. (2016). Development of Mathematical Models and Application of the Modified Gompertz Model for Designing Batch Biogas Reactors. Waste Biomass-Valorization.

[28] Taheri E., Amin M.M., Fatehizadeh A., Rezakazemi M., Aminabhavi T.M. (2021). Artificial intelligence modeling to predict transmembrane pressure in anaerobic membrane bioreactor-sequencing batch reactor during biohydrogen production. J. Environ. Manag..

[29] Ashok M., Kumar M. (2020). Anaerobic Membrane Reactors for Biohydrogen Production.

[30] Ngo H.H., Khan M.A., Guo W., Liu Y., Zhang X., Li J., Wang J. (2020). Energy Production in Anaerobic Membrane Bioreactors: Opportunities and Challenges.

[31] Lee K.-S., Lin P.-J., Fangchiang K., Chang J.-S. (2007). Continuous hydrogen production by anaerobic mixed microflora using a hollow-fiber microfiltration membrane bioreactor. Int. J. Hydrogen Energy.

[32] Yoon S.-H. (2015). Membrane Bioreactor Processes.

[33] Navaratna D., Shu L., Baskaran K., Jegatheesan V. (2012). Model development and parameter estimation for a hybrid submerged membrane bioreactor treating Ametryn. Bioresour. Technol..

[34] Ran N., Sharon-Gojman R., Larsson S., Gillor O., Mauter M.S., Herzberg M. (2022). Unraveling pH Effects on Ultrafiltration Membrane Fouling by Extracellular Polymeric Substances: Adsorption and Conformation Analyzed with Localized Surface Plasmon Resonance. Environ. Sci. Technol..

[35] Wang Z., Wu Z., Tang S. (2009). Extracellular polymeric substances (EPS) properties and their effects on membrane fouling in a submerged membrane bioreactor. Water Res..

[36] Lew B., Tarre S., Beliavski M., Dosoretz C., Green M. (2009). Anaerobic membrane bioreactor (AnMBR) for domestic wastewater treatment. Desalination.

[37] Ozgun H., Dereli R.K., Ersahin M.E., Kinaci C., Spanjers H., van Lier J.B. (2013). A review of anaerobic membrane bioreactors for municipal wastewater treatment: Integration options, limitations and expectations. Sep. Purif. Technol..

[38] Nilusha R.T., Wang T., Wang H., Yu D., Zhang J., Wei Y. (2020). Optimization of in situ backwashing frequency for stable operation of anaerobic ceramic membrane bioreactor. Processes.

[39] Qiu C., Zheng Y., Zheng J., Liu Y., Xie C., Sun L. (2016). Mesophilic and Thermophilic Biohydrogen Production from Xylose at Various Initial pH and Substrate Concentrations with Microflora Community Analysis. Energy Fuels.

[40] Qin Y., Cheng H., Li Y.-Y. (2022). Sustainable anaerobic technologies for biogas and biohythane production. Biomass, Biofuels, Biochemicals: Circular Bioe-Conomy: Technologies for Waste Remediation.

[41] Shen L., Bagley D.M., Liss S.N. (2009). Effect of organic loading rate on fermentative hydrogen production from continuous stirred tank and membrane bioreactors. Int. J. Hydrogen Energy.

[42] Lee D.-Y., Li Y.-Y., Noike T. (2010). Influence of solids retention time on continuous H_2_ production using membrane bioreactor. Int. J. Hydrogen Energy.

[43] Noblecourt A., Christophe G., Larroche C., Santa-Catalina G., Trably E., Fontanille P. (2017). High hydrogen production rate in a submerged membrane anaerobic bioreactor. Int. J. Hydrogen Energy.

[44] Lee D., Xu K., Kobayashi T., Li Y., Inamori Y. (2014). Effect of organic loading rate on continuous hydrogen production from food waste in submerged anaerobic membrane bioreactor. Int. J. Hydrogen Energy.

[45] Oh S.-E., Iyer P., Bruns M.A., Logan B.E. (2004). Biological hydrogen production using a membrane bioreactor. Biotechnol. Bioeng..

[46] Lee D.-Y., Li Y.-Y., Noike T., Cha G.-C. (2008). Behavior of extracellular polymers and bio-fouling during hydrogen fermentation with a membrane bioreactor. J. Membr. Sci..

[47] Kim D., Lee D., Kim M. (2011). Enhanced biohydrogen production from tofu residue by acid/base pretreatment and sewage sludge addition. Int. J. Hydrogen Energy.

[48] Venetsaneas N., Antonopoulou G., Stamatelatou K., Kornaros M., Lyberatos G. (2009). Using cheese whey for hydrogen and methane generation in a two-stage continuous process with alternative pH controlling approaches. Bioresour. Technol..

[49] Palomo-Briones R., Razo-Flores E., Bernet N., Trably E. (2017). Dark-fermentative biohydrogen pathways and microbial networks in continuous stirred tank reactors: Novel insights on their control. Appl. Energy.

